# ΔMST and the Regulation of Cardiac CSE and OTR Expression in Trauma and Hemorrhage

**DOI:** 10.3390/antiox10020233

**Published:** 2021-02-03

**Authors:** Britta Trautwein, Tamara Merz, Nicole Denoix, Csaba Szabo, Enrico Calzia, Peter Radermacher, Oscar McCook

**Affiliations:** 1 Institute for Anesthesiological Pathophysiology and Process Engineering, Ulm University Medical Center, 89081 Um, Germany; britta@lukaschewski.de (B.T.); tamara.merz@uni-ulm.de (T.M.); nicole.denoix@uni-ulm.de (N.D.); enrico.calzia@uni-ulm.de (E.C.); peter.radermacher@uni-ulm.de (P.R.); 2 Clinic for Psychosomatic Medicine and Psychotherapy, Ulm University Medical Center, 89070 Ulm, Germany; 3 Department of Science and Medicine, University of Fribourg, 1700 Fribourg, Switzerland; csaba.szabo@unifr.ch

**Keywords:** hydrogen sulfide, oxytocin, mitochondria, early life stress, psychosomatic, anxiolytic

## Abstract

Genetic deletion of 3-mercaptopyruvate sulfurtransferase (MST) is known to result in hypertension and cardiac hypertrophy in older mice, and is associated with increased anxiety-like behaviors. Endogenous hydrogen sulfide (H_2_S) produced by MST in the mitochondria is also known to be involved in physiological and cellular bioenergetics, and its dysfunction associated with depressive behavior and increased cardiovascular morbidity. Interestingly, early life stress has been shown to lead to a significant loss of cystathionine-γ-lyase (CSE) and oxytocin receptor (OTR) expression in the heart. Thus, we were interested in testing the hypothesis of whether genetic MST mutation (ΔMST) would affect cardiac CSE and OTR expression and affect the mitochondrial respiration in a clinically relevant, resuscitated, mouse model of trauma and hemorrhagic shock. In ΔMST mice, we found a reduction of CSE and OTR in both the naive as well as injured state, in contrast to the wild type (wt) controls. Interestingly, the ΔMST showed a different complex IV response to injury than the wt controls, although our claims are based on the non-demonstrated assumption that naive wt and naive ΔMST mice have comparable complex IV activity. Finally, hemorrhagic shock led to a reduction of CSE and OTR, confirming previous results in the injured mouse heart. To date, the exact mechanisms of the cardiac interaction between H_2_S and OT are not clear, but they point the way to potential cardioprotective therapies.

## 1. Introduction

The neuroendocrine oxytocin (OT) system and the gasotransmitter hydrogen sulfide (H_2_S), endogenously produced by cystathionine β-lyase (CSE), cystathionine γ-synthase (CBS), and 3-mercaptopyruvate-sulfurtransferase (MST), have been shown to have parallel roles in the heart in response to trauma. OT and H_2_S are relevant in models of both psychological and physical trauma, displaying cardio-protective effects [1].

Genetic deletion (ko) of MST has resulted in hypertension and cardiac hypertrophy, at least in aged mice [2]; furthermore, it has been associated with increased anxiety-like behaviors [3]. We investigated the effects of a genetic MST mutation (ΔMST) in a resuscitated mouse model of traumatic-hemorrhagic shock, and found no significant differences in hemodynamics, gas exchange, metabolism, acid base status, or survival between ΔMST and the respective wild type groups [4].

Hydrogen sulfide (H_2_S) is reported to be cardioprotective in ischemia reperfusion injury [5,6,7,8], and endogenous H_2_S produced by MST localized to the mitochondria has been reported to support basal, physiological, and cellular bioenergetics function [9]. In response to osmotic balance stress, H_2_S can stimulate OT release from magnocellular neurons of the paraventricular nuclei (PVN) in the hypothalamic regulation of blood volume and pressure. Hemorrhagic shock (HS) is the highest acute blood volume change in trauma, and can lead to hypoxic events in the brain [10]. In addition, Amini-Khoei et al. demonstrated that depressive-like behavior caused by early life stress (ELS) is associated with mitochondrial dysfunction, and that administration of oxytocin (OT) improves mitochondrial function and behavior [11]. ELS is known to increase cardiovascular morbidity [12,13], and moreover, ELS leads to a significant loss of CSE and oxytocin receptor (OTR) expression in the heart [14]. These results in the ELS model are similar to the findings we report in an acute-on-chronic trauma model: the interaction of the H_2_S and OT system in cardiovascular injury. Cardiac OTR was significantly downregulated, and the downregulation was even more pronounced in mice with genetic CSE deletion, but it could be restored by exogenous H_2_S administration (GYY4137) [15]. Furthermore, naive CSE knock out (ko) mice had lower levels of OTR [1], and similarly, naive mice with a genetic deletion of OTR presented with a reduction of CSE expression [14]. Psychological stress can reportedly dysregulate OTR expression [16], and trauma leads to cardiovascular co-morbidities, which increase morbidity and mortality in intensive care patients [1,17,18]. Trauma can be a result of either (or both) a deep emotional pain, and/or a life-threatening event (psychological) or a physiological injury or impact against the body (physical). Recent research has indicated that both physical [15] and psychological [14] trauma share physiological properties [1,19].

The dearth of published data on the role of MST in the heart and our interest in the role of CSE and OTR in both psychological and physical trauma motivated us to evaluate post hoc cardiac samples from a previous experiment [4] and concurrent naive animals. Our goal was to focus on the role of OTR and CSE, due to their interaction in fluid shifts and in psychological stress, which is relevant in light of the anxiolytic role of MST. Therefore, this study tested the hypothesis of whether the ΔMST phenotype affects the cardiac CSE/OTR expression, and whether this coincides with alterations of mitochondrial respiration.

## 2. Materials and Methods

The study protocol was authorized by the federal authorities for animal research of the Regierungspräsidium Tübingen (approved animal experimentation number: 1190), Baden-Württemberg, Germany, and the Animal Care Committee of the University of Ulm, Baden-Württemberg, Germany. Experiments were performed in adherence with the National Institutes of Health Guidelines on the Use of Laboratory Animals and the European Union “Directive 2010/63 EU on the protection of animals used for scientific purposes”. C57BL/6J mice were received from Charles River laboratories Germany (Sulzbach, Germany), and ΔMST as previously reported [4]. The generation of ΔMST mice has previously been described in detail [20]. Briefly, ΔMST mice were generated by a disruption of the MST gene by gene trapping, using the Omnibank gene trap vector containing a dominant splice acceptor site that integrated upstream of the MST protein coding region in the mouse genome in C57Bl6 embryonic stem cells. The resulting animals are characterized by reduced MST mRNA expression in many tissues (lung, muscle, testis) [20] and reduced MST protein levels in the lung, spleen, and pancreas [20,21]. MST protein levels in the kidney and liver are not affected by the mutation [20,21], and to the best of our knowledge, the effects of this mutation on MST expression in the heart have not been investigated to date.

### 2.1. Polytrauma Model

At the beginning of the experiment, mice were anesthetized with inhaled sevoflurane (Sevorane, Abbott, London, UK) and subcutaneous buprenorphine (Temgesic, Reckitt Benckiser, Slough, UK). Then, a sham procedure or blunt chest trauma (Txt), generated by a midthoracal, single blast wave, as described previously, was induced [4]. Afterwards, all mice received intraperitoneal ketamine (Ketanest-S, Pfizer, New York, NY, USA), midazolam (Midazolam-ratiopharm, Ratiopharm, Ulm, Germany), and fentanyl (Fentanyl-hameln, Hameln Pharma Plus GmbH, Hameln, Germany), and were placed in a mouse intensive care unit as previously described [4,22,23], comprising lung-protective mechanical ventilation, hemodynamic monitoring and management, and closed-loop temperature control. All animals underwent 1 h of hemorrhagic shock (HS), induced by blood withdrawal adjusted to either the maximum volume (30 μL/g body weight) or a mean arterial blood pressure (MAP) of 35 mmHg [23]. Afterwards, mice were resuscitated with shed-blood, colloid fluids (hydroxyethyl starch, Tetraspan, Braun Medical), or, if necessary, continuous norepinephrine infusions to maintain MAP ≥ 50 mmHg [22]. Systemic hemodynamic parameters and static thoracopulmonary compliance were recorded hourly; blood gas tensions, acid–base status, glycemia, and lactatemia were assessed at the end of the 4 h period of resuscitation [4]. At the end of the experiment, animals were exsanguinated, and the blood and organs were sampled. The number of animals per group was as follows: wild type hemorrhagic shock (wt HS) = 10, wt HS with blunt chest trauma (wt HS Txt) = 10, ΔMST HS = 8, ΔMST HS + Txt = 8. Naive animals (wt: *n* = 9, ΔMST: *n* = 9) were anesthetized with sevoflurane (Sevorane, Abbott, London, UK) and buprenorphine (Temgesic, Reckitt Benckiser); mid-line laparotomy was performed, and animals were sacrificed via venous exsanguination [15]. The heart was harvested, and the apex placed in ice-cold Custodiol for mitochondrial respiration measurements. The remaining heart was cut transversally and formalin-fixed for immunohistochemistry [4,15].

### 2.2. Mitochondrial Respiration

Mitochondrial respiratory capacity was measured using high-resolution respirometry with a Clark electrode-based system (Oxygraph-2k respirometer, Oroboros instruments Corp., Innsbruck, Austria), as previously described [23,24]. Immediately post mortem, heart tissue was mechanically homogenized in the respiration medium (Mir05/EGTA = 0.5 mM, MgCl_2_·6H_2_O = 3 mM, lactobionic acid = 60 mM, taurine = 20 mM, KH_2_PO_4_ = 10 mM, HEPES = 20 mM, sucrose = 110 mM, and bovine serum albumin = 1 g/L) and 1.5 mg of tissue were transferred to the Oxygraph chamber. A defined sequence of substrates or inhibitors were added to assess various states of mitochondrial respiratory capacity. The activity of complex I was measured after the addition of 10 mM pyruvate, 10 mM glutamate, 5 mM malate, and 5 mM ADP; in order to assess the maximum oxidative phosphorylation (OxPhos), 1 mM octanoyl-carnitine and 10 mM succinate were added. Leak compensation flux was assessed after the inhibition of ATP-synthase by 2.5 μM oligomycin; the maximum respiratory activity of the electron transfer system in the uncoupled state (ETS) was measured after titrating carbonyl cyanide-4-(trifluoromethoxy)phenylhydrazone (FCCP; final concentration = 1.5 μM). The activity of complex IV was determined by adding 2 mM ascorbate and 0.5 mM *N*,*N*,*N*’,*N*’-Tetramethyl-*p*-phenylenediamine dihydrochloride (TMPD) [4,22].

### 2.3. Immunohistochemistry

**Figure 1:**
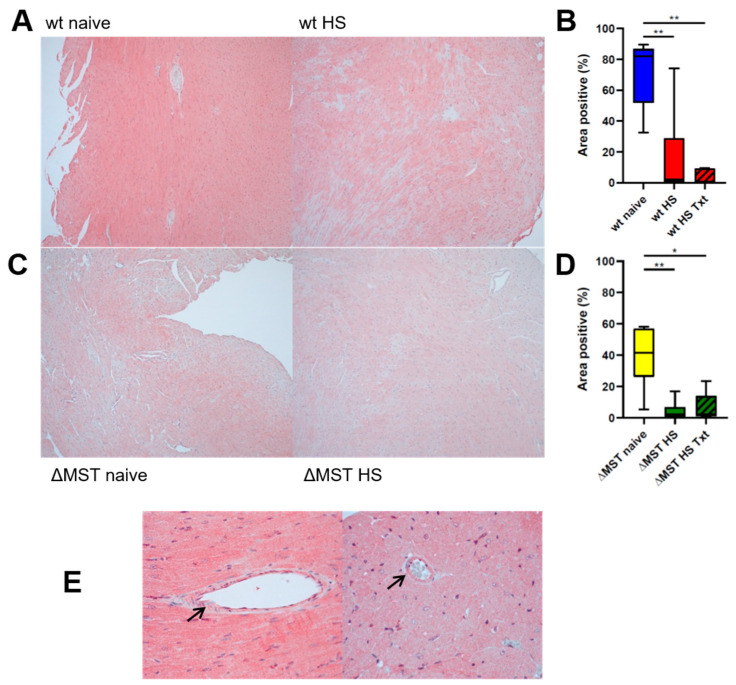
Cardiac 3-mercaptopyruvate sulfurtransferase (MST) protein expression. (**A**) Exemplary pictures for MST expression in cardiac tissue from naive and injured wild-type (wt) mice (magnification = 10×). (**B**) Quantification of MST expression (wt naive *n* = 8, wt hemorrhagic shock (HS) *n* = 10, wt HS blunt chest trauma (Txt) *n* = 7). (**C**) Exemplary pictures for MST expression in cardiac tissue from naive and injured genetic MST mutation (ΔMST) mice (magnification = 10×). (**D**) Quantification of MST expression (ΔMST naive *n* = 8, ΔMST HS *n* = 8, ΔMST HS Txt *n* = 8). Boxplots depict the median and interquartile range; whiskers represent the minimum and maximum values. * *p* < 0.05, ** *p* < 0.01. (**E**) Exemplary pictures of MST expression in large (left = 40×) and small (right = 64×) coronary vessels (arrows).

We specifically decided to prioritize immunohistochemistry (IHC) analysis over Western blot (WB), because WB would only limit the amount of information that could be had, in that it would obviate the topographical landscape and specific localization of the protein of interest would be lost (e.g., vascular MST expression depicted in Figure 1A,C,E). We have previously shown that the quantification of protein via IHC directly correlates with the quantification by Western blot (*p* = 0.001, *R*^2^ = 0.76) [25]. In Wigger et al., protein quantification via both IHC and WB also revealed the same result [14]. Quantification of protein expression via IHC can be inaccurate when using fluorescent antibodies, which is why we rely on a red chromogen and permanent mount, which prevents decaying of the signal, as would be the case with fluorescence. Furthermore, an often-neglected point is that it is well-known that WB is open to contamination by whole blood products containing the H_2_S endogenous enzymes [26] and OTR [27]. Thus, all methods that require tissue homogenization like WB are prone to generate inaccurate results due to potential contamination, unless previously perfused, which is very rarely done.

As previously described [15] hearts were formalin-fixed, dehydrated, embedded in paraffin, and then cut into 3 µm sections. The slides were deparaffinized, rehydrated, and heat-induced antigen retrieval using citrate buffer pH 6.0 was performed. Slides were then blocked with 10% goat serum. To assess the expression of CSE, OTR, and MST in the heart, the following primary antibodies were used: anti-CSE (rabbit polyclonal; Proteintech, 12217-1-AP, Rosemont, IL, USA), anti-OTR (rabbit polyclonal; Proteintech, 23045-1-AP), and anti-MST (rabbit polyclonal; Sigma, HPA001240, Saint Louis, MO, USA) [14,15]. Primary antibodies were detected by a secondary antibody (goat-anti-rabbit IgG conjugated to alkaline phosphatase; Jackson ImmunoResearch, Cambridge, UK). Antibodies were visualized with a red chromogen (Dako REAL Detection System Chromogen Red, Dako, Glostrup, Denmark), and tissue was counterstained with Mayers hematoxylin (Sigma). The staining was evaluated with the Zeiss Axio Imager A1 microscope and the Axio Vision 4.8 software (CSE, OTR) and Zeiss Zen 3.0 (MST) (Zeiss, Oberkochen, Germany). Two distinct 800,000 µm^2^ regions were quantified and presented as densitometric sum red or area % [15].

### 2.4. Statistical Analysis

All data are presented as medians (25th and 75th percentile) unless stated otherwise. Mitochondrial respiratory capacity was analyzed with a two-way ANOVA, followed by a post-hoc Tukey test. For CSE and OTR immunohistochemistry, normal distribution was confirmed with the Kolmogorov–Smirnov test, and differences between groups were analyzed using an ordinary one-way ANOVA and a post-hoc Holm–Sidak test. For MST, normal data distribution was excluded with the Kolmogorov–Smirnov test, and differences between groups were analyzed using the Kruskal–Wallis ANOVA on ranks, followed by a post-hoc Dunn test for two-tailed multiple comparisons. Quantitative relations between mitochondrial function, CSE, and OTR expression were assessed by measuring the Pearson coefficient of correlation. For this purpose, data from individual animals were pooled, no matter the group assignment. A result of *p* < 0.05 was considered as statistically significant. Quantitative graphical presentations and statistical analyses were performed with GraphPad Prism 8 (Graphpad Software Inc., San Diego, CA, USA).

## 3. Results

We detected MST in the heart and coronary vasculature of our ΔMST mice (see Figure 1A,E). MST expression was significantly reduced after injury in both wt and ΔMST animals compared to the respective naive group (see Figure 1B,D). There was no difference between naive wt and ΔMST mice with Kruskal–Wallis analysis of all six groups. When comparing only these two groups in a Mann–Whitney rank sum test, the MST reduction in ΔMST animals reaches statistical significance (*p* = 0.01).

**Figure 2:**
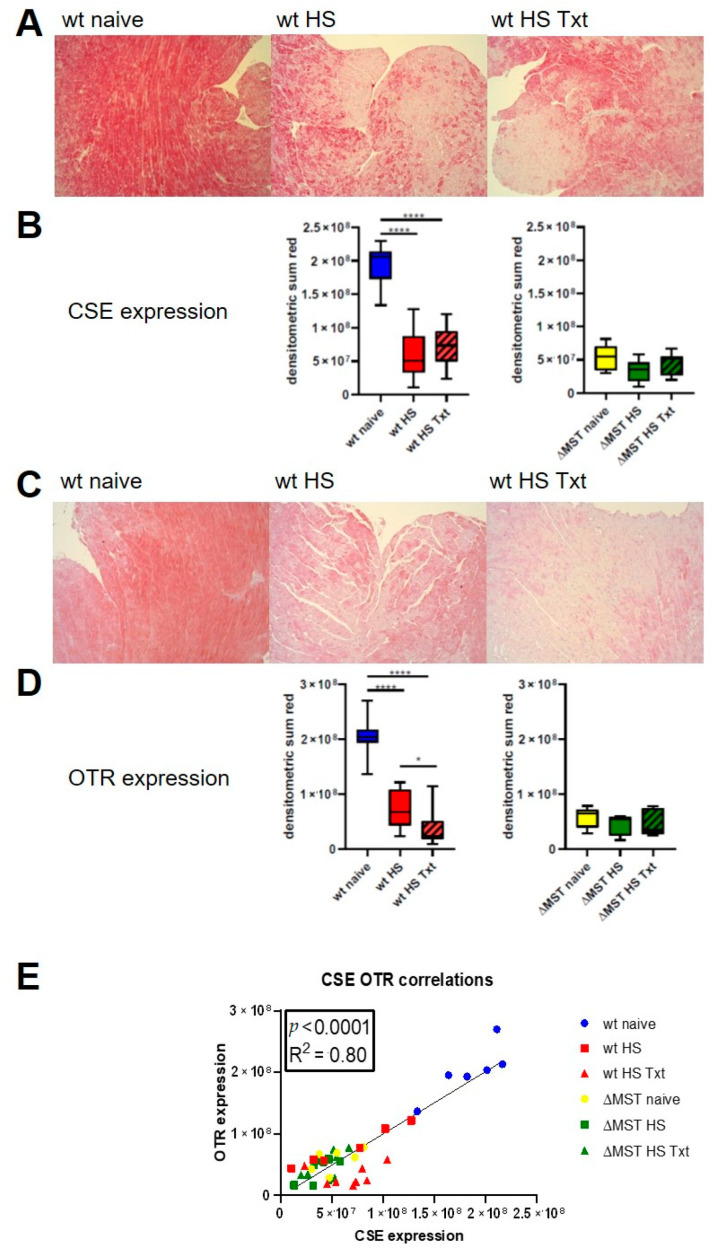
Cardiac cystathionine β-lyase (CSE) and oxytocin receptor (OTR) protein expression. (**A**) exemplary pictures for CSE expression in cardiac tissue from naive wt, wt HS, and wt HS Txt (magnification = 10×). (**B**) Quantification of CSE expression (wt naive *n* = 9, wt HS *n* = 10, wt HS Txt *n* = 9, ΔMST naive *n* = 9, ΔMST HS *n* = 8, ΔMST HS Txt *n* = 7). Boxplots depict the median and interquartile range, and whiskers represent the minimum and maximum values. **** *p* < 0.0001. (**C**) Exemplary pictures for OTR expression in cardiac tissue from naive wt, wt HS, and wt HS Txt (magnification = 10×). (**D**) Quantification of OTR expression (wt naive *n* = 9, wt HS *n* = 10, wt HS Txt *n* = 10, ΔMST naive *n* = 6, ΔMST HS *n* = 8, ΔMST HS Txt *n* = 7). Boxplots depict the median and interquartile range, and whiskers represent the minimum and maximum values. * *p* < 0.05, **** *p* < 0.0001. (**E**) Direct linear relationship between CSE and OTR protein expression in wt, ΔMST naive, and injured mouse hearts.

CSE and OTR were constitutively expressed in cardiomyocytes of naive animals (see Figure 2A,C, left panel). In wt animals, HS and HS + Txt significantly reduced expression of cardiac CSE and OTR compared to naïve animals. ΔMST animals had significantly lower CSE and OTR expression already in the naive state, and no further reduction in MST expression after injury was observed (see Figure 2A–D). The ΔMST groups all had reduced CSE and OTR expression compared to naive wt. There was no difference in CSE and OTR expression between ΔMST and wt after trauma (see Figure 2A–D). There was a direct, linear relationship between CSE and OTR expression in all animals, independent of the genetic background or injury (see Figure 2E).

**Figure 3:**
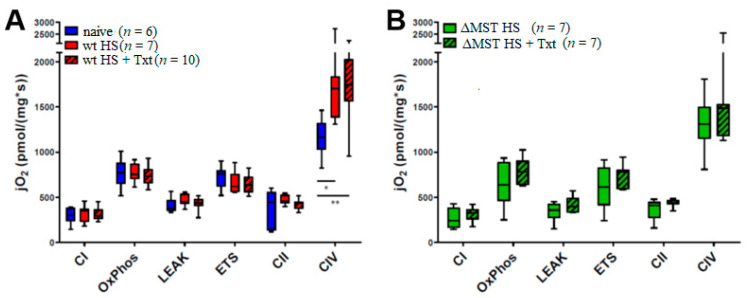
Mitochondrial respiratory capacity in the heart. (**A**) Wt animals. (**B**) ΔMST animals. jO2: oxygen flux, CI: mitochondrial oxygen consumption after addition of substrates for the stimulation of complex I, OxPhos: maximum respiratory capacity in the coupled state, LEAK: mitochondrial oxygen consumption after inhibition of ATP synthase in the coupled state, ETS: maximum respiratory capacity in the uncoupled state, CII: mitochondrial oxygen consumption depending on the activity of complex II after inhibition of complex I, CIV: oxygen consumption after stimulation of complex IV. Boxplots depict the median and interquartile range, and whiskers represent the minimum and maximum values. ** *p* < 0.01, * *p* < 0.05.

In mechanically permeabilized heart tissue, the mitochondrial activity of complex IV was significantly elevated in both wt HS and wt HS Txt trauma groups compared to naive wt animals. ΔMST HS and ΔMST HS + Txt animals had similar complex IV activity levels to the wt naive animals. No significant intergroup differences could be shown in the remaining states of mitochondrial function (see Figure 3).

## 4. Discussion

The main findings of this study were that (1) ΔMST mice had lower levels of MST in the heart than wt; (2) there was a downregulation of cardiac CSE and OTR expression in naïve ΔMST when compared to wt mice; (3) hemorrhagic shock induced (a) a cardiac mitochondrial upregulation in complex IV activity in wt animals and (b) a decrease of myocardial CSE and OTR expression in wt mice; (4) there was linear correlation between cardiac CSE and OTR expression, and that correlation holds true after injury.

Interestingly, MST was detected in the heart and coronary vasculature (see Figure 1C) of both wt and ΔMST naive mice. However, ΔMST mice presented with lower MST expression than the wt littermates. While both HS alone and the combination of HS and Txt further reduced cardiac MST, this difference between wt and ΔMST was abolished by the challenge of HS with(out) Txt.

The naive ΔMST had lower CSE and OTR expression when compared to the wt mice. In contrast to our findings in the ΔMST mice, Peleli et al., using a global MST-ko model found no change in the expression of the endogenous CSE and CBS enzymes [2]. The difference in expression pattern may be attributed to the fact that we used a mutated MST mouse model in comparison to Peleli’s global MST-ko. Nevertheless, Ahmad et al. using the same global MST-ko as Peleli et al., found lower endogenous CBS activity than in the wt animals in the liver [28], suggesting compartmentalization and varied regulation of the endogenous enzymes in different organs. Trauma induced a significant downregulation of CSE and OTR expression in the wt mouse heart upon hemorrhagic shock, but there was no additional loss of these enzymes in the ΔMST mice. Moreover, there was a significant direct linear relation between the of expression of CSE and OTR proteins, independent of the genotype of the mice (wt vs. ΔMST) and injury (see Figure 2E). 

This begs the question of what mechanistically was at play in the ΔMST naive mice that led to the reduced CSE and OTR expression in the heart. Furthermore, it is not immediately apparent why there are reported differences in the expression pattern of the endogenous enzymes in these models, while it may in part be due to the different injuries evaluated (i.e., HS [4], myocardial ischemia/reperfusion (MIR) [2], and burn [28]). It may also be pointing to a very complex interaction of the endogenous enzymes in their response to injury. In fact, in an earlier study comparing genetically modified strains of the endogenous H_2_S enzymes (CSE-ko, CBS partial ko, and ΔMST mice), Ahmad et al. show inherent and organ-specific variability in the genetically modified strains’ response to lipopolysaccharide (LPS) challenge [21]. Even though the expression patterns in the four organs evaluated (lung, kidney, liver, and spleen) were (mostly) similar, the few differences were striking: while in the ΔMST mice LPS increased CBS in the kidney only, it enhanced CBS expression in the spleen and lung in the wt mice, in contrast to the liver and kidney in the CSE-ko mice.

This is an intriguing variety of responses in the genetically modified strains, and suggests a very complex relationship among the three endogenous H_2_S-producing enzymes. In fact, ambivalent results are reported from the same group: on the one hand, the loss of MST conferred protection in MIR [2]; on the other hand, authors showed in a heart failure model that MST impaired mitochondrial respiration, which was ultimately associated with reduced exercise performance and increased cardiac dysfunction in their model [29].

In our study, in wt mice, the mitochondrial activity of complex IV was significantly elevated in both wt HS and HS Txt trauma groups when compared to naive animals, whereas the complex IV activity in ΔMST groups in response to injury was similar to wt naive levels. We had previously reported that our data did not indicate compromised respiratory activity in any of the investigated tissues of the trauma groups [4]. When contrasting these results in the trauma groups to the wt naive state, as was done here, we were then able to clearly recognize a significant trauma-related compensatory mechanism at play in complex IV in wt animals (see Figure 3). No significant intergroup differences could be shown in the remaining states of mitochondrial function.

We did not assess any possible behavioral effects of the ΔMST genotype, but the reduction of both CSE and OTR in the naive ΔMST mice may be related to the anxiolytic effects of MST reported by Nagahara et al. [3]. Nevertheless, Wigger et al. reported a similar loss of both CSE and OTR in the adult hearts of neonatal mice exposed to chronic, long-term maternal separation [14], an early life stress (ELS) model of psychosocial trauma. The loss of cardiac protein expression in response to psychological trauma is comparable to the effects of physical trauma reported here and previously [15]. Interestingly, the effects of psychosocial trauma were dependent on the stress dose: in the study from Wigger et al., short-term separation stress (STSS) had the opposite effect as long-term separation stress (LTSS) on cardiac OTR expression, upregulating it instead of downregulating it, which is speculated to mediate stress resilience in response to STSS. In contrast, LTSS rather seems to be associated with stress-induced vulnerability [14]. It is interesting to note that the ΔMST phenotype in the naive state leads to a similar loss of CSE and OTR as long-term ELS [2,3]. Similarly, both LTSS and a lack of MST are associated with increased anxiety and depressive-like behavior, mediated by alterations at the brain level [3,14]. Thus, the naive ΔMST animals are characterized by lower CSE and OTR expression in the heart, which coincides with a pattern seen in both psychological and physical trauma, suggesting an important, yet not fully resolved, role of MST in the context of stress-induced cardiovascular disease. 

Furthermore, the H_2_S and oxytocin system are both reported to be involved in mitochondrial functioning. The literature on exact mechanisms remains limited, but several studies suggest a direct interaction between oxytocin and mitochondria affecting mitochondrial bioenergetics [30] in, e.g., ischemia-reperfusion-injury [31] and early-life-stress [11]. We cannot say for certain what the implications of the apparent inability to upregulate complex IV activity in ΔMST in response to injury are, since our observations are based on the non-demonstrated assumption that naive wt and naive ΔMST mice have comparable complex IV activity; nor can we say whether this is regulated by the loss of either MST, CSE, OTR, or a combination thereof. Nevertheless, our findings confirm previously published studies suggesting an interaction of the H_2_S and oxytocin system [1,14,15]. Because H_2_S and oxytocin are considered to be cardioprotective [32,33,34], loss of the aforementioned proteins points to cardiovascular dysfunction. We recently showed the mutual interaction of H_2_S and the neuroendocrine oxytocin systems in cardiac injury [15]. OTR and CSE are constitutively expressed in the heart [15,33] and are reported to be cardioprotective [32,33,35,36,37]. CSE is the most important producer of endogenous H_2_S in the cardiovascular system [38]. In recent studies on resuscitated, co-morbid septic pigs displaying a similar reduction of cardiac output as patients with coronary artery disease, we were able to show reduced tissue CSE expression in the coronary artery [34], kidney [25], and heart [32], which was associated with increased troponin levels and CSE mRNA (compensating for reduced tissue protein expression) and reduced cardiac OTR expression [39]. In addition, Wang et al. propose that H_2_S and OT are able to both act via nitric oxide (NO) regulation, and that CSE might be able to mediate cardioprotection by OTR upregulation and activation of the reperfusion injury salvage kinase (RISK) pathway [40]. 

Coletti et al. showed, in a model of fluid shift, that water deprivation increased endogenous H_2_S production, and that exogenous H_2_S administration (Na_2_S) stimulated cerebral oxytocin release by inhibiting the NO system [41]. More recently, H_2_S, triggered by acute hyperosmolality, was described as a positive regulator of OT, in contrast to NO, which played the role of a negative neuroendocrine modulator of OT [42]. Both H_2_S and OT are also known as vasoactive mediators, and are therefore implicated in regulating circulating blood volume, blood pressure [43,44,45,46,47,48,49,50,51,52] and the heart rate [1,53,54]. Our results confirm our hypothesis that HS, a pronounced fluid shift, leads to a downregulation of CSE and OTR in the heart (see Figure 2B,D). Cardiac OTR and CSE directly correlated in wt and ΔMST animals in the naive state, as well as after injury (see Figure 2E). Although in this study we have no data for their expression in the brain, in a porcine model of HS we were able to co-localize the proteins in the hypothalamus and the corresponding magnocellular neurons [1], where they are reported to interact in response to fluid shifts [41]. The effects of ΔMST on the regulation of cerebral levels of CSE and OTR expression warrant further investigations. In retrospect, a limitation of this study is that we did not evaluate the CBS expression, which could provide an interesting piece of the puzzle and should be evaluated in future studies.

## 5. Conclusions

In a clinically relevant, resuscitated mouse model, naive and injured ΔMST mice had a reduction of cardiac CSE and OTR expression. Injured ΔMST mice had similar levels of mitochondrial complex IV activity to wt naive animals, whereas in injured wt animals higher complex IV activity was observed as an adaptive response. Hemorrhagic shock, a dramatic fluid shift, led to cardiac CSE and OTR downregulation in wt animals, confirming similar previous results in the injured mouse heart [14,15]. Additional confirmation of their interaction is the fact that their expression was directly correlated in animals in both the naive state as well as post-injury, even though the exact mechanisms of an interaction between the H_2_S and OT system have yet to be clarified. Previously, we reported that exogenous H_2_S administration (GYY4137) mediated an upregulation of cardiac OTR expression, which was accompanied by restored blood glucose levels and mean arterial pressure [15]. Taken together with the findings presented here, this study suggests that exogenous H_2_S administration may be a therapeutic option to mediate cardioprotection from both psychological and physical trauma by OTR upregulation.

## Data Availability

Data is contained within the article.

## Abbreviations

ANOVA	Analysis of Variance
CBS	Cystathionine-β-synthase
CI	Mitochondrial oxygen consumption after addition of substrates for the stimulation of complex I
CII	Mitochondrial oxygen consumption depending on the activity of complex II after inhibition of complex I
CIV	Oxygen consumption after stimulation of complex IV
CSE	Cystathionine-γ-lyase
ΔMST	MST mutation
ELS	Early-life stress
ETS	Maximum respiratory capacity in the uncoupled state
FCCP	Carbonyl cyanide-4-(trifluoromethoxy)phenylhydrazone
HS	Hemorrhagic shock
H_2_S	Hydrogen sulfide
IHC	Immunohistochemistry
jO_2_	Oxygen flux
ko	Genetic deletion/knock-out
LEAK	Mitochondrial oxygen consumption after inhibition of ATP synthase in the coupled state
LPS	Lipopolysaccharide
LTSS	Long-term separation stress
MAP	Mean arterial pressure
MIR	Myocardial ischemia/reperfusion
MST	3-mercaptopyruvate sulfurtransferase
NO	Nitric oxide
OT	Oxytocin
OTR	Oxytocin receptor
OxPhos	Maximum respiratory capacity in the coupled state
STSS	Short-term separation stress
TMPD	*N*,*N*,*N*’,*N*’-Tetramethyl-*p*-phenylenediamine dihydrochloride
Txt	Blunt chest trauma
WB	Western blot
wt	Wild type
